# Adjuvant therapy for gastric cancer – has the standard changed?

**DOI:** 10.1054/bjoc.2000.1657

**Published:** 2001-04

**Authors:** J W Valle

**Affiliations:** Department of Medical Oncology, Christie Hospital NHS Trust, Wilmslow Road, Manchester, M20 4BX, UK


British Journal of Cancer (2001) 84(7), 875–877
© 2001 Cancer Research Campaign
doi: 10.1054/ bjoc.2000.1657, available online at http://www.idealibrary.com on IDEAL®

http://www.bjcancer.com

Editorial

### Adjuvant therapy for gastric cancer – has the standard changed?

**JW Valle**

Senior Lecturer/ Honorary Consultant in Medical Oncology, Department of Medical Oncology, Christie Hospital NHS Trust, Wilmslow Road, Manchester M20 4BX, UK

'Post-operative chemotherapy cannot be considered as a standard adjuvant treatment for gastric cancer.' This was the conclusion of a meta-analysis in 1993 of 11 trials including 2096 patients in which post-operative adjuvant chemotherapy was compared to surgery alone (Hermans et al, 1993). The authors found a non-significant survival advantage (odds-ratio (OR) 0.88, 95% confidence interval (CI) 0.78–1.08). However, this figure was later adjusted to include data from studies omitted in error to a statistically significant result (OR 0.82, 95% CI: 0.68–0.97) (Hermans and Bonenkamp, 1994).

A more recent meta-analysis evaluated 13 randomized controlled trials performed between 1966–99 (Earle and Maroun, 1999). All of the studies had a surgery-alone control arm versus post-operative chemotherapy of varying regimens in non-Asian patients. The overall crude odds-ratio for death in the treated group (preserving stratification by study, but not adjusting for prognostic variables) was 0.80 (95% CI: 0.66–0.97). The magnitude of the effect was smaller for trials with more than 5 years follow-up although a trend towards benefit was maintained suggesting a long-term effect, not just delaying relapse. In addition, this effect was most marked in lymph node-positive disease by sub-group analysis.

Since this meta-analysis 3 further studies have been reported suggesting a benefit from adjuvant chemotherapy or chemo-radiotherapy after surgery for gastric cancer. Cirera et al (1999) randomized 156 patients who had undergone resection of stage III disease to receive either no further treatment (control arm) or chemotherapy comprised of a single dose of mitomycin-C (20 mg/$m^2$ on day 1) followed by oral tegafur (400 mg bd, starting on day 30 and continued for 3 months). After a median follow-up of 37 months, there was a significant difference in overall survival (median survival 74 vs. 29 months, $P = 0.04$) and disease-free survival (63 vs. 22 months, $P = 0.01$) favouring the chemotherapy-arm. The 2-year and 5-year survival figures were 72% vs. 58% and 56% vs. 36%, respectively, also in favour of the chemotherapy-arm. The estimated hazard ratio for mortality in the treatment group compared to the control arm was 0.60 (95% CI: 0.39–0.93).

Neri et al (2001) in this edition, publish an update of their previously presented study (Neri et al, 1996). This update provides 5-year follow-up data (previously an interim analysis after 36 months) on 137 patients. All patients had lymph node-positive disease and were randomized to either surgery alone or surgery followed by chemotherapy with epidoxorubicin 75 mg/$m^2$ (day 1) and leucovorin 200 mg/$m^2$ plus 5-FU 450 mg/$m^2$ (days 1–3), repeated every 3 weeks for 7 months. 88% of patients randomized to receive chemotherapy received all planned cycles of treatment. The median survival of patients having surgery alone was 18 months compared to 31 months in the chemotherapy group ($P < 0.01$), with an estimated hazard ratio for mortality of 1.96 for the control arm compared to the chemotherapy-arm (95% CI: 1.32–2.92).

The results of Intergroup Study INT-0116 were presented at the American Society of Clinical Oncology annual meeting this year (Macdonald et al, 2000). In this large study, 603 patients were randomized to either surgery alone or surgery followed by adjuvant chemo-radiotherapy. This consisted of one cycle of leucovorin/5-FU (20 mg/$m^2$ and 425 mg/$m^2$, days 1–5) followed by 4500 cGy in 25 fractions given with leucovorin/5-FU (20 mg/$m^2$ and 400 mg/$m^2$) on days 1–4 and 23–25 of radiotherapy. This was followed, a month later, by 2 further cycles of leucovorin/5-FU (20 mg/$m^2$ and 425 mg/$m^2$, days 1–5, given at monthly intervals). After a median follow-up of 3.3 years, there was a significantly improved overall survival (3-year survival: 52% vs. 41%, $P = 0.03$) and disease-free survival (3-year DFS: 49% vs. 32%, $P = 0.001$), both in favour of the chemo-radiotherapy arm. The median survival was 27 months in the surgery alone arm vs. 42 months in the combined modality arm (estimated mortality hazard ratio of 1.28).

Are we to conclude, therefore, that adjuvant treatment is now the standard of care in gastric cancer? The patient population assessed in the 3 studies outlined above is not uniform. In the Spanish study (Cirera et al, 1999) all patients had stage III disease, which would have included node-negative patients (see Table 1). Only 7% of control patients compared to 20% of chemotherapy patients had N0 disease. However, these differences did not reach statistical significance ($P = 0.07$) and the beneficial effects of chemotherapy remained after adjustment for nodal infiltration. Neri et al selected node-positive patients only and the numbers of patients with N1 and N2 disease was balanced between the control and chemotherapy arms. However, patients with lymph node-positive disease can range from stage IB (T1N1M0) to stage IV (T4, N2–3) according to AJCC criteria (AJCC, 1997). The intergroup Study, INT-0116, randomized patients with stages IB through to IV, including patients with both lymph node-positive and -negative disease. 85% of patients had nodal metastases and the distribution of N0 disease was balanced with 16% in the surgery-alone arm and 14% in the chemotherapy arm. There were too few patients ($n = 36$, 18 in each arm) in the stage IB sub-group to make any firm conclusions about these patients who, even without treatment have a relatively good prognosis.

The extent of surgery was also different in the 3 studies. Cirera et al recruited patients who had surgical margins free from tumour and who had undergone an extended lymphadenectomy (R2 resection). This may account for their excellent 5-year survival figures

**Table 1** AJCC group staging for gastric cancer

| | Group staging | | |
|---|---|---|---|
| Stage 0 | Tis | N0 | M0 |
| Stage IA | T1 | N0 | M0 |
| Stage IB | T1 | N1 | M0 |
| | T2 | N0 | M0 |
| Stage II | T1 | N2 | M0 |
| | T2 | N1 | M0 |
| | T3 | N0 | M0 |
| Stage IIIA | T2 | N2 | M0 |
| | T3 | N1 | M0 |
| | T4 | N0 | M0 |
| Stage IIIB | T3 | N2 | M0 |
| Stage IV | T4 | N1 | M0 |
| | T1 | N3 | M0 |
| | T2 | N3 | M0 |
| | T3 | N3 | M0 |
| | T4 | N2 | M0 |
| | T4 | N3 | M0 |
| | Any T | Any N | M1 |

(36% control arm, 56% chemotherapy arm) although the median follow-up is relatively short (37 months) and mature data is awaited. In the Italian study (Neri et al, 2000) 13% of patients in the control arm and 15% of patients in the chemotherapy arm underwent an R2 resection, the remainder undergoing either R1A or R1B resections. However, in their multivariate analysis, the type of surgery was not found to be a significant prognostic factor ($P > 0.75$).

Is this finding significant? Two surgical studies in which a total of 989 patients who had undergone a R0 resection and were then randomized to either a D1 or D2 lymph node dissection have failed to show a survival benefit from extended lymph node dissection at 3 years. In the first, the 3-year survival was 56% (D1 resection) vs. 58% (D2 resection) among 589 patients randomized in the Dutch Gastric Cancer Trial (Bonenkamp et al, 1999). The Medical Research Council, in the second study showed a 50% vs. 57% 3-year survival (D1 vs. D2 respectively) among 400 patients (Cuschieri et al, 1999). It must be noted that surgical morbidity and mortality were higher for the D2-treated patients. In the INT-0116 study, 10% of patients had a D2 resection, 36% a D1 resection and 54% did not conform to a D1 resection (<D1). However, in the chemo-radiotherapy arm the 3-year survival was 52%, approximating the 3-year survival following D1 resection in the 2 surgical studies raising the possibility that chemo-radiotherapy is making up for sub-optimal surgery. This adds weight to the view that surgery for gastric cancer should be performed by experienced surgeons aiming for a D1 resection. Further studies assessing the value of chemotherapy or chemo-radiotherapy in patients who undergo adequate surgery are warranted given the positive findings of Neri et al and Cirera et al.

In the Spanish study (Cirera et al, 1999) the most frequent sites of relapse occurred at local and peritoneal sites (48% controls vs. 54% treatment group). Patients in the INT-0116 study had a reduced local relapse rate in the treatment arm (19% vs. 29% controls) and a higher distant relapse rate (35% vs. 18% controls). Is the role of radiotherapy, therefore, to complement surgery when resection has been sub-optimal, and would this benefit be lost if all patients underwent at least a D1 resection? This will only be made clear by future studies. Unfortunately, Neri et al do not provide data for sites of relapse.

At what price to the patient is this survival benefit being achieved? The mitomycin-C and tegafur combination was particularly well tolerated with only one documented grade III toxicity (neurocerebellar). Grade II toxicity included nausea and vomiting ($n$ = 5) and leucopenia ($n$ = 1), mucositis ($n$ = 1) and diarrhoea ($n$ = 1). The epidoxorubicin, leucovorin and 5-FU combination was more toxic with grade III/IV toxicity as follows: mucositis (12%), diarrhoea (8.7%), leucopenia (8.7%), anaemia (4.3%) and thrombocytopenia (2.9%). Despite this, no patients required hospitalization. Chemo-radiotherapy was described as tolerable but was much more toxic with grade III toxicity in 41% overall (haematological 54%, gastro-intestinal 33%, infection 6% and neurological 4%) and grade IV toxicity in 32% of cases. There were 3 (1%) toxic deaths. In order to minimize the toxicity of this regimen, all radiotherapy fields were reviewed and, following this review, radiotherapy planning was changed in 34% of cases. It is estimated that had this review not been performed, serious, potentially life-threatening toxicity may have occurred in up to 10% of cases (Kelsen, 2000).

It seems that the body of evidence is changing since the conclusion of the 1993 meta-analysis. Several positive studies advocate the use of adjuvant chemotherapy or chemo-radiotherapy. Patients with lymph node-positive disease appear to benefit (although evidence is lacking for patients with stage IB disease) as do patients with stage III disease (whether lymph node-positive or -negative). Improving the numbers of patients who undergo a D1-lymph node resection may improve overall survival and may also negate the need for the radiotherapy modality in chemo-radiotherapy. Given the heterogeneity of the regimens used in these studies, the optimum regimen for use has still not been defined. Additionally, the effect of other approaches such as neo-adjuvant chemotherapy or chemo-radiotherapy and the role of newer generation cytotoxic agents (such as docetaxel and irinotecan, either alone or in combination with current agents) has yet to be determined. This group of patients should continue to be encouraged to participate in well-designed randomized controlled trials, some of which are already underway.

### REFERENCES

AJCC (1997) Stomach. In: *American Joint Commitee on Cancer: Staging Manual*, pp. 71–76. Lippincott-Raven Publishers: Philadelphia, PA

Bonenkamp JJ, Hermans J, Sasako M and van de Velde CJ (1999) Extended lymph-node dissection for gastric cancer. Dutch Gastric Cancer Group [see comments]. *N Engl J Med* **340**: 908–914

Cirera L, Balil A, Batiste Alentorn E, Tusquets I, Cardona T, Arcusa A, Jolis L, Saigi E, Guasch I, Badia A and Boleda M (1999) Randomized clinical trial of adjuvant mitomycin plus tegafur in patients with resected stage III gastric cancer. *J Clin Oncol* **17**: 3810–3815

Cuschieri A, Weeden S, Fielding J, Bancewicz J, Craven J, Joypaul V, Sydes M and Fayers P (1999) Patient survival after D1 and D2 resections for gastric cancer: long-term results of the MRC randomized surgical trial. Surgical Co-operative Group. *Br J Cancer* **79**: 1522–1530

Earle CC and Maroun JA (1999) Adjuvant chemotherapy after curative resection for gastric cancer in non-Asian patients: revisiting a meta-analysis of randomised trials. *Eur J Cancer* **35**: 1059–1064

Hermans J and Bonenkamp H (1994) In reply (letter). *J Clin Oncol* **12**: 879–880

Hermans J, Bonenkamp JJ, Boon MC, Bunt AM, Ohyama S, Sasako M and Van de Velde CJ (1993) Adjuvant therapy after curative resection for gastric cancer: meta-analysis of randomized trials [see comments]. *J Clin Oncol* **11**: 1441–1447

Kelsen D (2000) Postoperative adjuvant chemoradiation therapy for patients with resected gastric cancer: Intergroup 116. *J Clin Oncol* **18**: 32s–34s

*© 2001 Cancer Research Campaign*

Macdonald JS, Smalley S, Benedetti J, Estes N, Haller DG, Ajani JA, Gunderson LL, Jessup M and Martenson JA (2000) Postoperative combined radiation and chemotherapy improves disease-free survival (DFS) and overall survival (OS) in resected adenocarcinoma of the stomach and G. E. junction. Results of Intergroup Study INT-0116 (SWOG 9008). *Proc Am Soc Clin Oncol* **19**: 1a

Neri B, de Leonardis V, Romano S, Andreoli F, Pernice LM, Bruno L, Borrelli D, Valeri A, Fabbroni S, Intini C and Cini G (1996) Adjuvant chemotherapy after gastric resection in node-positive cancer patients: a multicentre randomised study. *Br J Cancer* **73**: 549–552

Neri B, Cini G, Andreoli F, Boffi B, Francesconi D, Mazzanti R, Medi F, Mercatelli A, Romano S, Siliani L, Tarquini R and Moretti R (2000) Randomised trial of adjuvant chemotherapy versus control after curative resection for gastric cancer: 5-year follow-up. *Br J Cancer* Current volume

*© 2001 Cancer Research Campaign*

